# Unveiling a Coalescing Catastrophe: Pre-pyloric Perforation Co-existing With Sigmoid Volvulus in a Middle-Aged Patient

**DOI:** 10.7759/cureus.55042

**Published:** 2024-02-27

**Authors:** Mihir Patil, Pankaj Gharde

**Affiliations:** 1 General Surgery, Jawaharlal Nehru Medical College, Datta Meghe Institute of Higher Education and Research, Wardha, IND

**Keywords:** modified graham's patch, pyloric perforation, intestinal perforation, intestinal detorsion, dilated sigmoid loops, acute intestinal obstruction

## Abstract

Sigmoid volvulus is a common cause of colonic obstruction worldwide and constitutes the majority of all cases of colonic volvulus. It is more prevalent in those who are older than 70 years. The sigmoid colon, an S-shaped portion of the large intestine, is susceptible to this condition due to its redundancy and mobile nature. Treatment involves endoscopic detorsion with sigmoidectomy. Laparoscopic surgery has been found to be useful in terms of reduced morbidity, blood loss, analgesics, and hospital stay; contrarily, surgical management has been found to be associated with reduced recurrence. Early diagnosis is crucial to prevent complications and recurrence rates.

Gastroduodenal perforation, whether spontaneous or traumatic, is predominantly associated with peptic ulcer disease. Specifically, the majority of perforated peptic ulcers are attributed to *Helicobacter pylori* infection. The presence of perforation as a comorbidity complicates surgical management, particularly when the patient has a history of *H. pylori* infections, as evidenced in our case. Addressing these infections is crucial for optimizing treatment outcomes and reducing potential complications.

Laparoscopic surgery is popular due to its benefits and faster recovery periods, especially in the aged population. This is a case presentation of a 48-year-old male who presented at our tertiary care center with abdominal pain, multiple episodes of vomiting, obstipation, and abdominal distention. The patient was diagnosed with sigmoid volvulus with pre-pyloric perforation which was managed surgically by initial detorsion followed by sigmoidectomy with modified Graham's patch technique. He recovered well with no post-operative complications.

## Introduction

The sigmoid colon, the S-shaped portion of the large intestine, is susceptible to volvulus due to its redundancy and mobile nature. A sigmoid volvulus is a medical condition where the sigmoid colon, which is a part of the large intestine, twists upon itself. This twisting causes a blockage, leading to symptoms like severe abdominal pain, bloating, constipation, sometimes nausea or vomiting, and potential complications [1]. It requires immediate treatment to prevent complications like reduced blood flow leading to tissue damage due to the twisting of blood flow. Sigmoid volvulus typically arises due to a combination of factors involving the anatomy and function of the sigmoid colon [2]. Endoscopic detorsion is a preferred treatment in sigmoid volvulus patients with fewer complications, and sigmoid resection and anastomosis are recommended in complicated cases such as sigmoid volvulus patients with gangrenous colon. As per the available research, most of the non-complicated cases can be managed by detorsion with morbidity and mortality at 26.4% and 9.1%, respectively [3], but the chances of recurrence are reported as high as 67% [4]. Additionally, the decision of management of the volvulus by surgical or non-surgical means can be convoluted by the presence of bowel gangrene or perforation. Co-existing morbidities can greatly influence the course of treatments such as anastomosis, resection, and others as it might limit certain procedures. Gastroduodenal perforation, which can occur spontaneously or due to trauma, is predominantly associated with peptic ulcer disease. The majority of spontaneous perforations are attributed to peptic ulcers, with *Helicobacter pylori* infection being a significant contributing factor. This bacterium is a major risk factor in the development of peptic ulcers, alongside other factors such as excessive gastric acid secretion and lifestyle habits. Prompt diagnosis and appropriate management, including the eradication of *H. pylori*, are essential to prevent complications such as perforation. In cases of perforation, emergency medical intervention, often involving surgery, is necessary to repair the defect and prevent further complications [5]. A comparatively novel approach by minimally invasive laparoscopic surgery has been reported to be found useful in terms of reduced morbidity, blood loss, analgesics, and duration of hospital stay [6,7].

## Case presentation

**Figure 1:**
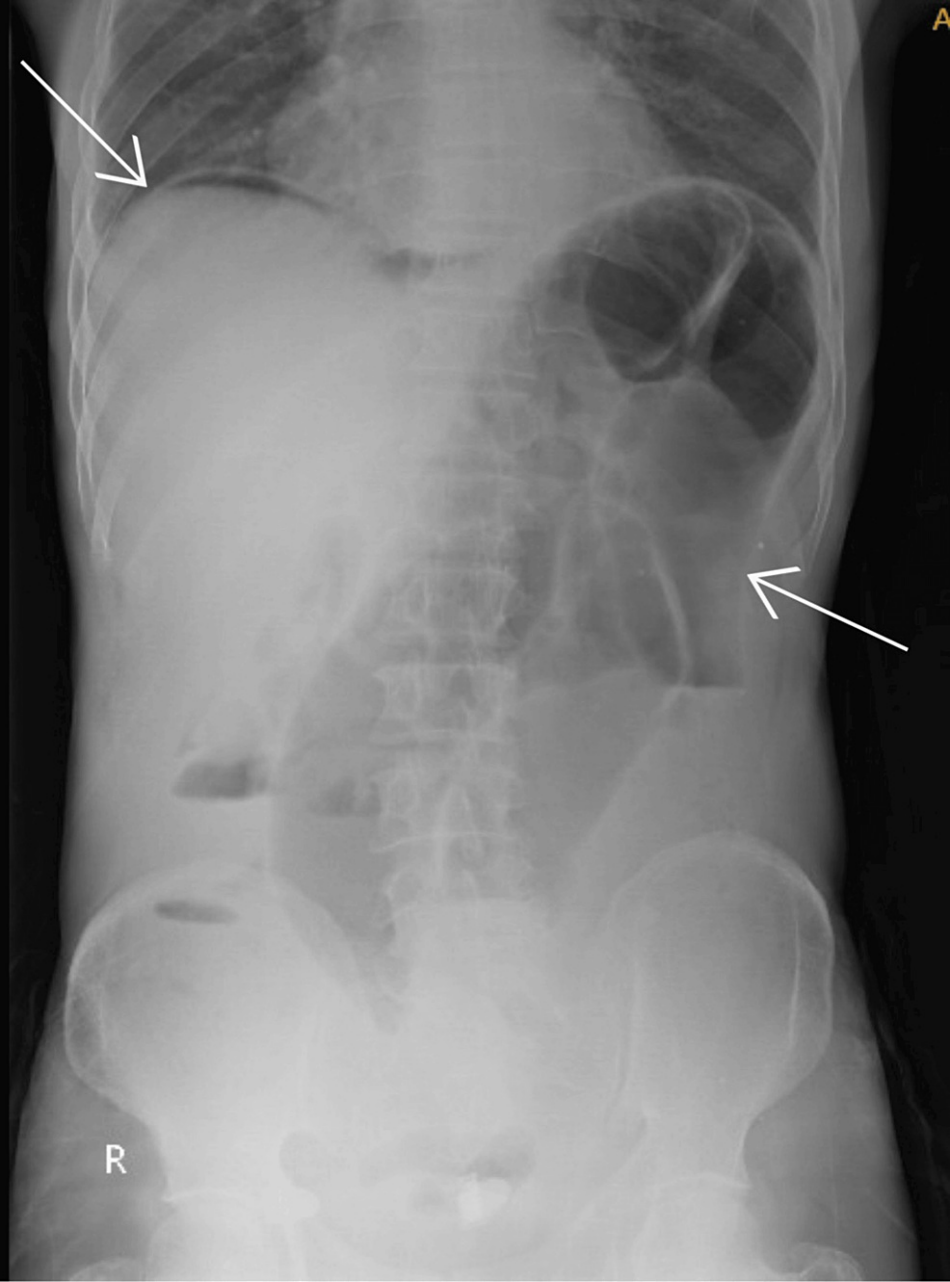
X-ray image suggestive of gas under the diaphragm and sigmoid volvulus

A 48-year-old male presented at our hospital with major complaints of abdominal distension, intermittent cramps, and obstipation for four days. The patient also had five to six episodes which were bilious in nature and were non-projectile for two days. He had a history of fever which was continuous throughout the day for one day and a history of *H. pylori* infection with gastritis six months ago for which he was started on medical management and later was a defaulter. Physical examination revealed the patient as poorly built and moderately nourished. Also, he had pallor and no icterus/cyanosis/clubbing/lymph adenopathy/edema. Vitals were observed as 128/minute pulse rate with borderline hypotension with 100/60 mmHg blood pressure, and the patient was febrile with a normal breathing pattern and dry buccal mucosa. Abdominal examination revealed a distended abdomen with guarding and rigidity. Tenderness was present all over the abdomen with no bowel sounds. On percussion, the abdomen was hypertympanic. Digital rectum examination showed an empty and collapsed rectum. Other physical examinations of the cardiovascular, respiratory, and central nervous systems were observed within normal limits. Radiological examination by X-ray was suggestive of gas under the diaphragm (suggestive of perforation) and a coffee bean sign suggestive of sigmoid volvulus (Figure 1).

**Figure 2:**
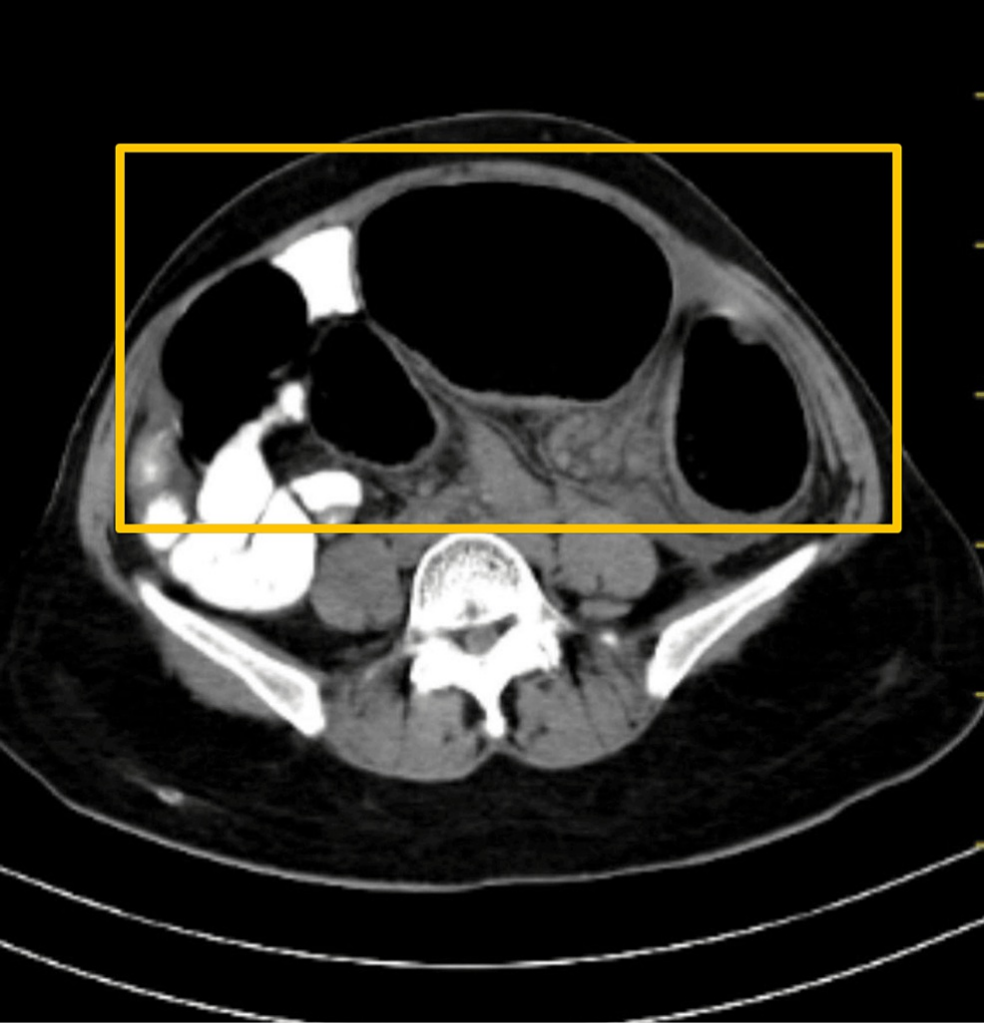
CT of the abdomen showing a dilated sigmoid loop, suggestive of acute intestinal obstruction

**Figure 3:**
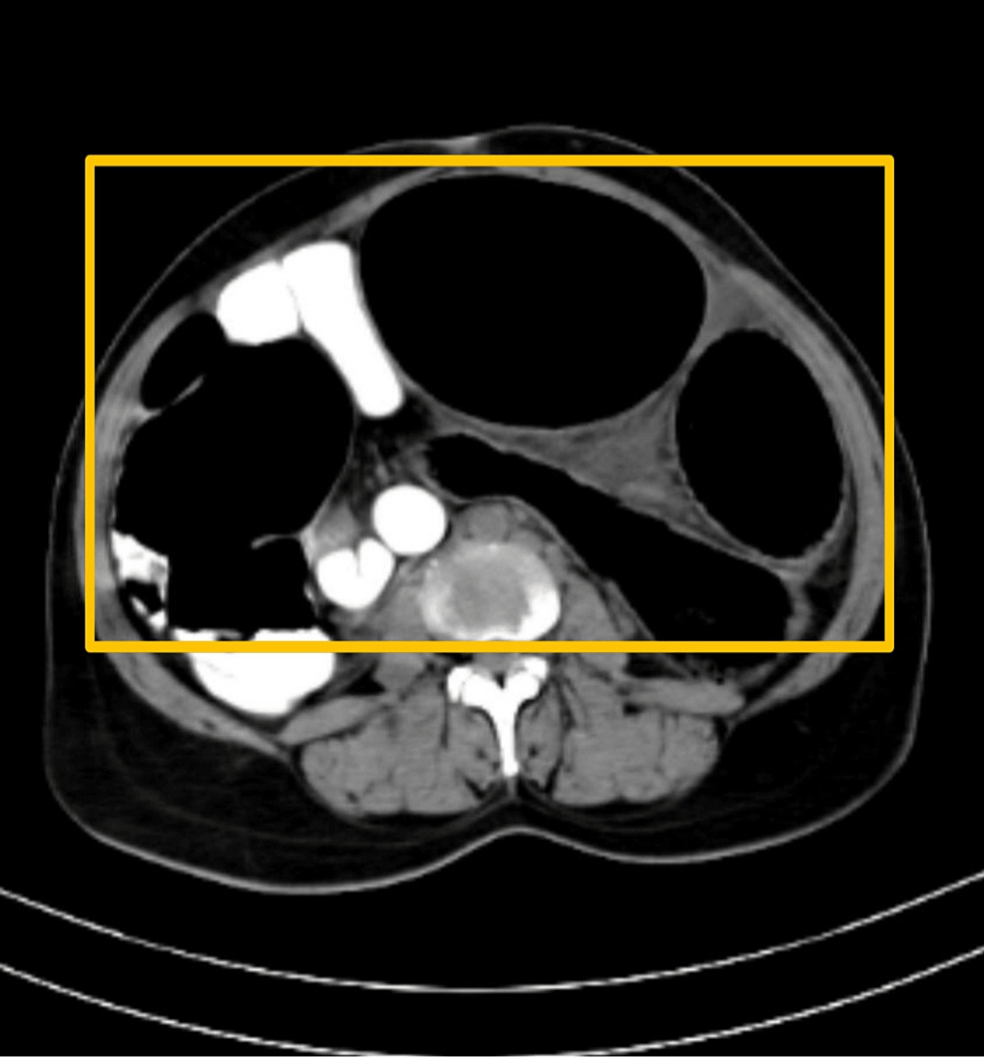
CT of the abdomen showing a dilated sigmoid loop likely to be sigmoid volvulus

**Figure 4:**
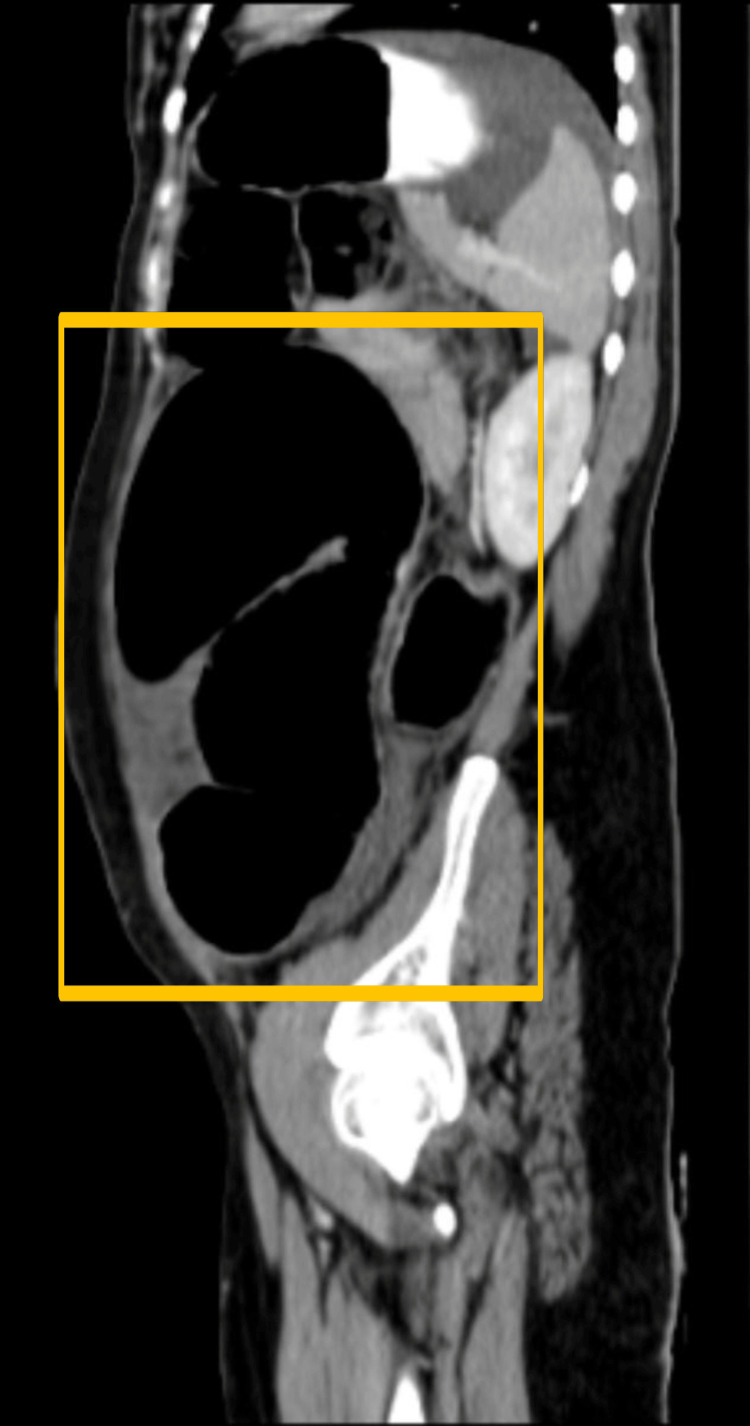
Sagittal section of the CT of the abdomen showing sigmoid volvulus

CT of the abdomen was carried out which showed a dilated large bowel loop likely to be a sigmoid volvulus with a distally collapsed bowel, distal to the transition point (Figure 2, Figure 3, and Figure 4).

Blood investigations of the patients are mentioned in Table 1.

**Table 1:** Lab investigations of the patient

Parameter	Value	Normal range
Hemoglobin	8.2 gm/dL	12.0-16.0 gm/dL
Total leucocyte count	23,000 cells/mm^3^	4,000-10,000 cells/mm^3^
Total platelet count	190,000 lakh/cu mm	1.5-4.1 lakh/cu mm
Urea	58 mg/dL	19-43 mg/dL
Creatinine	1.1 mg/dL	0.66-1.25 mg/dL
Sodium	132 mmol/L	135-145 mmol/L
Potassium	3.0 mmol/L	3.5-5.1 mmol/L
Alkaline phosphatase	200 U/L	38-126 U/L
Aspartate aminotransferase	163 U/L	17-59 U/L
Alanine aminotransferase	86 U/L	<50 U/L
Total protein	6 g/dL	6.0-8.3 g/dL
Albumin	3 g/dL	3.5-5 g/dL
Total bilirubin	1.0 mg/dL	0.2-1.3 mg/dL
Conjugated bilirubin	0.9 mg/dL	0.0-0.3 mg/dL
Unconjugated bilirubin	0.1 mg/dL	0.0-1.1 mg/dL

**Figure 5:**
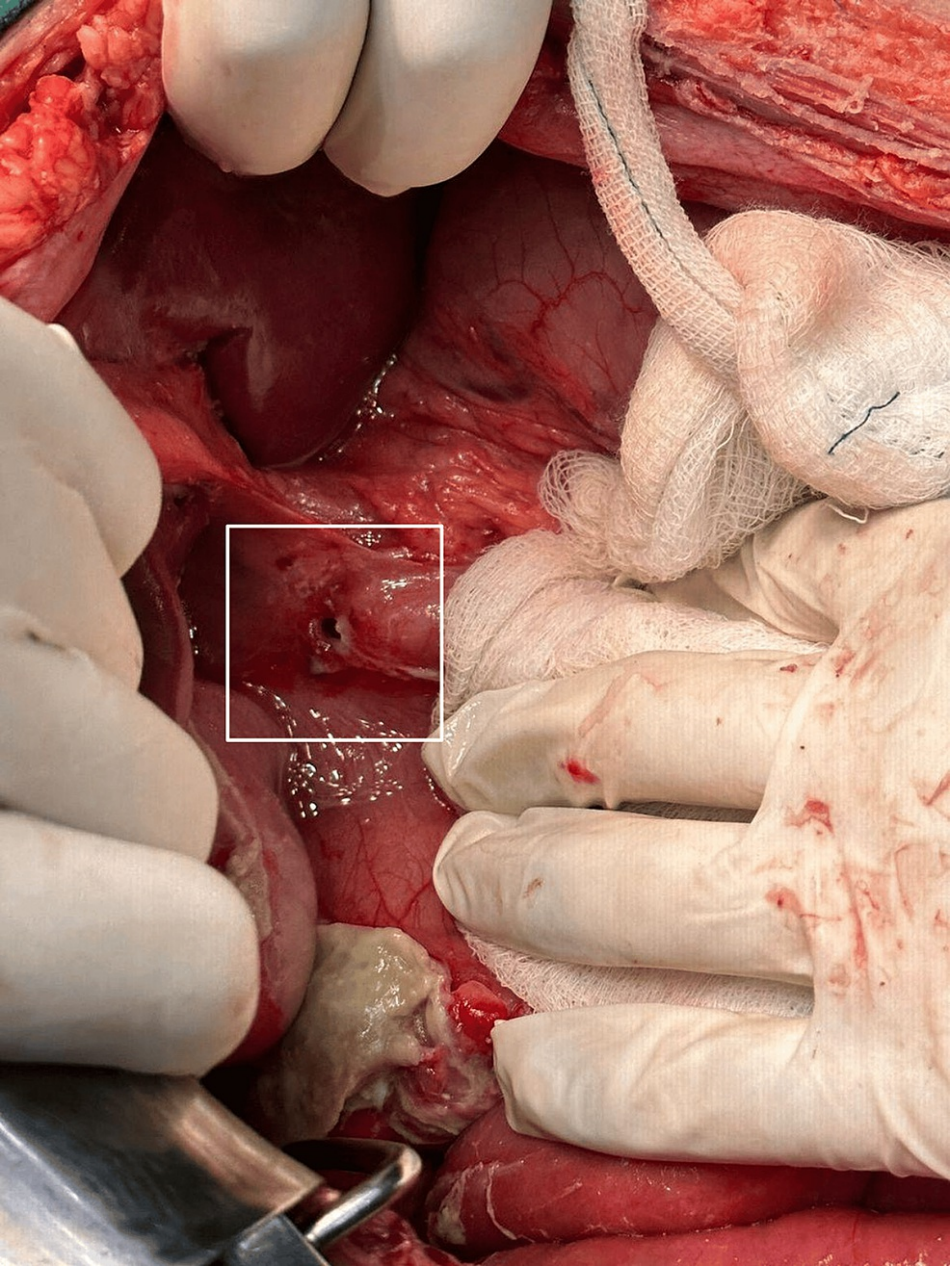
Perforation in the pre-pyloric region

**Figure 6:**
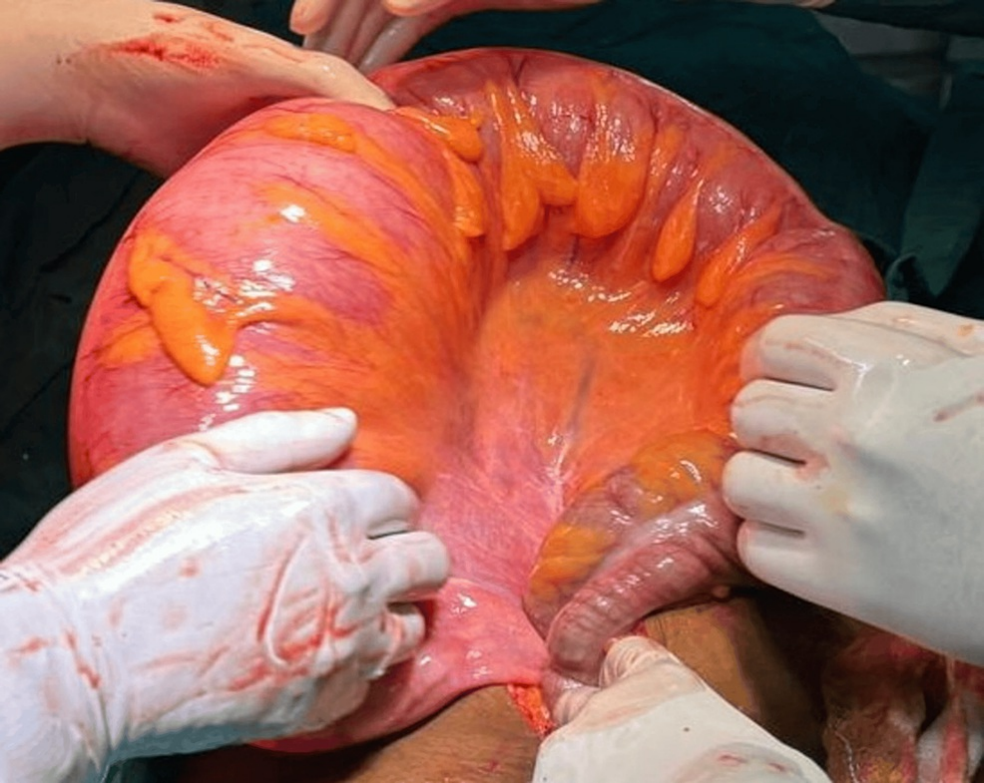
Dilated sigmoid colon (sigmoid volvulus)

The patient was admitted, intravenous fluid resuscitation was done, and vital signs were corrected with adequate urine output. Pre-operatively, he was transfused with one unit of packed red blood cell (PRBC) and was taken up for emergency exploratory laparotomy based on the impression of generalized peritonitis secondary to bowel perforation with sigmoid volvulus (Figure 5, Figure 6). 

**Figure 7:**
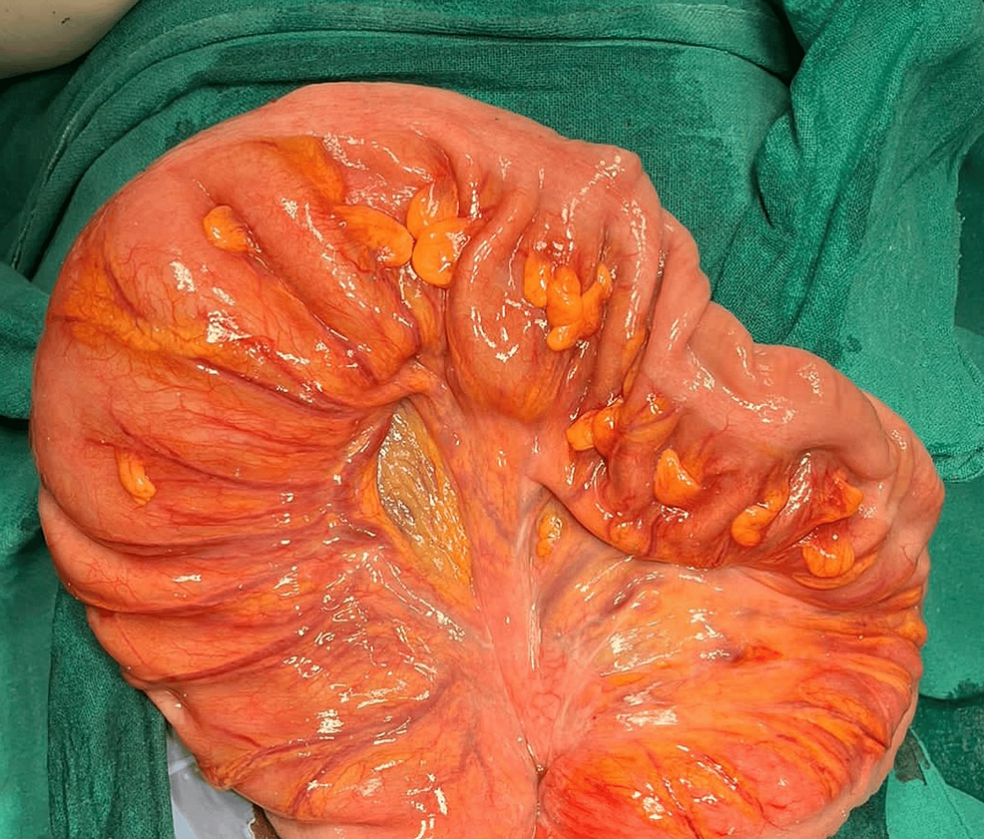
Resected volvulated segment specimen

**Figure 8:**
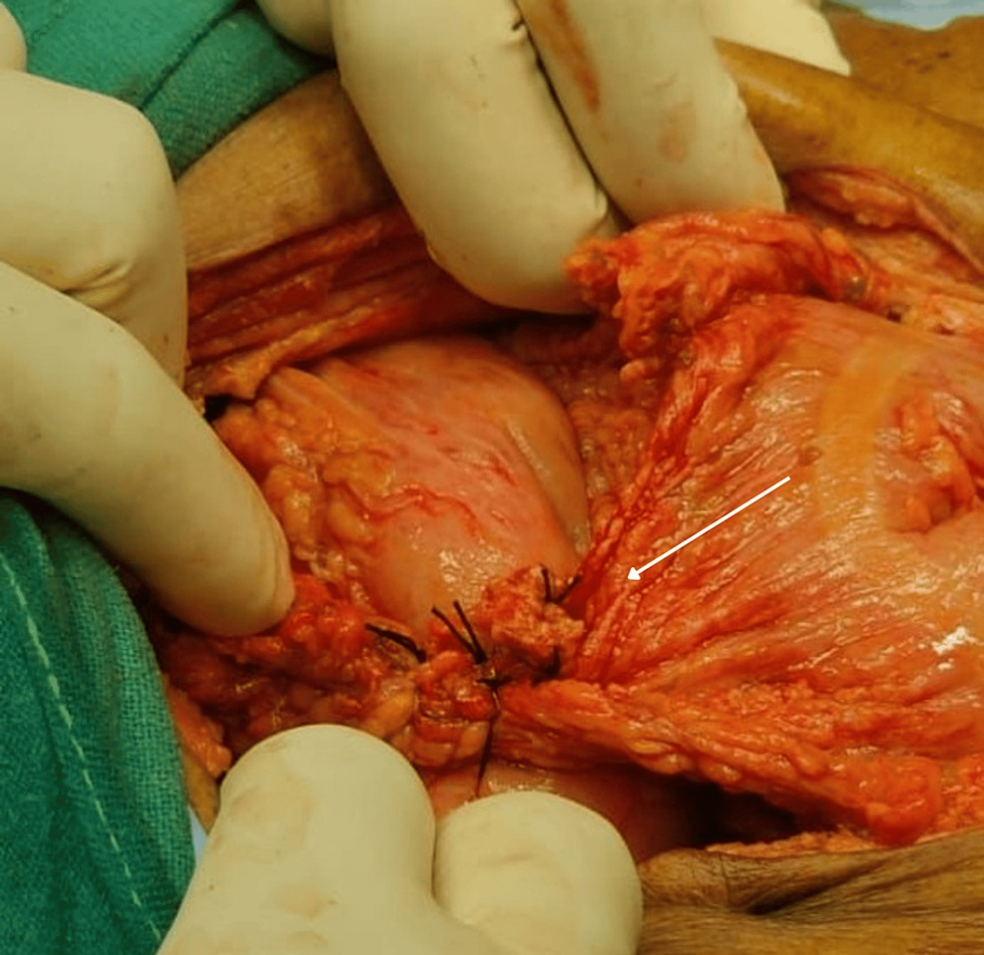
Modified Graham's patch repair done for pre-pyloric perforation

Midline exploratory laparotomy incision was done, and the abdomen was opened. Intra-operatively, about 150 mL of pus with gastric contents were drained, and a small perforation was noted at the pre-pyloric region. A 270º clockwise grossly volvulated sigmoid colon was noted. Distended large and small bowel loops were observed. Intra-operatively, the decision was made, and initial detorsion with sigmoidectomy was done as there were multiple small necrotic patches over the volvulated segment. Later, the pre-pyloric perforation was sealed and modified Graham's patch repair was done, a thorough wash was given, and hemostasis was achieved followed by the closure of the abdomen in layers (Figure 7, Figure 8).

The patient was extubated and shifted to the surgical ICU for post-operative care. Post-operatively, he was managed with antibiotics, analgesics, and supportive measures. Also, he passed flatus and stools after six days of surgery. The post-operative period was uneventful, and the patient was discharged after two weeks post-surgery.

## Discussion

One of the most common causes of colonic obstruction is sigmoid volvulus which consists of up to 50-90% of the total colonic volvulus in different parts of the world. It is most commonly observed in individuals >70 years of age with a higher incidence in pregnant females and a male predominance [3,4], which has been explained by the inability of the bowel to untwist due to inadequate force and muscle tiredness which is further aggregated by the production of gas and distended colon loop entrapped in the strong abdominal wall and enlarged uterus in males and females, respectively [8]. Some primary causes include anatomical predisposition, abnormal motility, previous abdominal surgeries, ageing, low-fiber diets, inadequate fluid intake, or habits that promote constipation which may potentially increase the risk of sigmoid volvulus [9]. Management of sigmoid volvulus primarily focuses on clearing the obstruction and decompression of the colon by varied methods such as endoscopic decompression, anastomosis, and intestinal resection which can be complicated due to the additional presence of intestinal perforation and gangrenous bowel.

There has been a reported rise in the incidence of pre-pyloric perforation in young adults which can be a matter of concern in the surgical management of the intestine for sigmoid volvulus, which has majorly been attributed to substance abuse, dyspepsia, cancer, *H. pylori* infection, and related causes. *H. pylori* infection can be managed by antibiotic recommendations based on the antibiotic susceptibility profile of the patient, proton pump inhibitors, and probiotics [10]. The co-existence of sigmoid volvulus and pre-pyloric perforation was observed in this case, which was assumed to be an outcome of the *H. pylori* infection in the patient six months back. Endoscopic intestinal decompression can be preferred in uncomplicated cases of sigmoid volvulus; however, surgical resection has been associated with decreased recurrence [11]. A research by Choi et al. reported majority of the patients of their study were relieved by non-surgical decompression, though recurrence was observed in patients without subsequent surgical correction, which was managed by Hartman's procedure on grounds of differential colonic diameter leading to better and efficient outcomes in such patients [12]. A systematic review suggested that two-point fixation yielded good outcomes and decreased recurrence as it helped avoid re-twisting as compared to single-point fixation [13]. This is the absolute last resort and is done in patients who are at very high risk for surgery.

The decision of mode of management of sigmoid volvulus is crucial, as the early recurrence rates reported by a large study of post-endoscopy decompression and surgical management were 4.9% and 0.6%, respectively [14]. Recommendations by the World Society of Emergency Surgery consensus guidelines on sigmoid volvulus management have suggested a detailed evaluation of blood parameters and physical examination with a focus on bowel ischemia, diagnostic abdominal radiographs for a coffee bean sign as a marker with CT imaging recommendation only in cases of doubted diagnosis, and endoscopic decompression as the first-line treatment in patients with no sign of perforation or intestinal ischemia, while patients with perforation or recurrence be managed by surgical resection. These guidelines also proposed a low level of evidence for sigmoid colectomy as a corrective procedure with a decreased rate of recurrences and marked non-resectional operative procedures equally effective [15]. The laparoscopic approach has gained popularity due to its benefits and faster recovery periods with a special focus on the aged population, and planned surgery can lead to a better outcome in this population subset [5,14-16]. Timely diagnosis is crucial in order to prevent complications such as gangrenous bowel, peritonitis, and even mortality. Early detection might be helpful and can prevent the need for colonic resection or anastomosis.

## Conclusions

Swift identification and management are imperative in cases of sigmoid volvulus to avert potential complications. Early radiological imaging aids in confirming the diagnosis, with CT imaging providing additional confirmation when necessary. While surgical resection of the sigmoid is often advised to prevent recurrence, endoscopic resection offers expedited recovery and reduced complications, particularly beneficial for the geriatric population due to its lower morbidity and post-operative concerns. Regarding gastroduodenal perforations, the majority arise spontaneously from peptic ulcer disease, warranting surgical intervention in almost all instances. Laparotomy with omental patch repair remains the standard approach, although laparoscopic surgery is increasingly favored due to its favorable outcomes.
